# Cellular and molecular effects of metronomic vinorelbine and 4-*O*-deacetylvinorelbine on human umbilical vein endothelial cells

**DOI:** 10.1097/CAD.0000000000000319

**Published:** 2016-02-01

**Authors:** Eirini Biziota, Evangelos Briasoulis, Leonidas Mavroeidis, Marios Marselos, Adrian L. Harris, Periklis Pappas

**Affiliations:** a Department of Pharmacology, School of Medicine; b Interscience Molecular Laboratory, Cancer Biobank Center; c Department of Haematology, Division of Medicine, School of Life Sciences, Ioannina, Greece; d Molecular Oncology Laboratories, Weatherall Institute of Molecular Medicine, John Radcliffe Hospital, University of Oxford, Oxford, UK

**Keywords:** human umbilical vein endothelial cell, interleukin-8, metronomic chemotherapy, nuclear factor-κB, peroxisome proliferator-activated receptor γ, vinorelbine

## Abstract

Metronomic oral vinorelbine (VRL; Navelbine) was shown in clinical trials to yield sustainable antitumor activity possibly through antiangiogenic mechanisms. We investigated the effects of protracted low-dose VRL on human umbilical vein endothelial cells, compared with a conventional chemotherapy model. Human umbilical vein endothelial cell cultures were treated with different concentrations of VRL (0.001 nmol/l to 1 mmol/l) for 4, 24 and 96 h. The effects of different drug concentrations on cell growth, cell cycle, apoptosis and expression of the angiogenesis-modulating genes *interleukin-8, cyclooxygenase-2, CD36 and peroxisome proliferator-activated receptor γ* were assessed using the metronomic or conventional chemotherapy model. Apoptosis and cell-cycle effects were assessed by flow cytometry. Gene expression was measured at the transcript level by quantitative reverse transcriptase-PCR, protein expression by immunoblotting and levels of proteins secreted in the cell medium by enzyme-linked immunosorbent assay. Activation of the nuclear factor-κB pathway was investigated by immunoblot analysis of cytosolic and nuclear protein extracts. The half-maximal inhibitory concentrations (IC_50_) of VRL at 96 h were four orders lower compared with those after a 24-h exposure (1.23 nmol/l vs. 32 mmol/l for VRL). Drug concentrations at high nanomolar levels and above, which are relevant to conventional pulsatile dosing of VRL, induced a dose-dependent and *nuclear factor-κB*-related increase in proangiogenic *interleukin-8* and *cyclooxygenase-2* and a decrease in the thrombospondin-1 receptor CD36 and *peroxisome proliferator-activated receptor γ* at mRNA and protein levels. In contrast, the opposite was evident with protracted picomolar to low nanomolar concentrations (metronomic dosing). Our data provide experimental support for metronomic VRL by showing that a protracted low dose outperforms pulsed high-dose administration in inducing antiangiogenic effects in proliferating human endothelial cells.

## Abstract 2

Supplemental Digital Content is available in the text.

## Background

Cytotoxic anticancer drugs are conventionally administered in a pulsatile manner at maximum tolerated doses with the aim of inducing the highest possible apoptosis of cancer cells. However, at these doses, they also affect healthy proliferating tissues and require treatment-free intervals to allow recovery from toxicities. These treatment gaps are known to facilitate repair of damaged endothelial and tumour cells and often to render most solid cancers resistant to these agents 1,2.

Metronomic chemotherapy is a novel dose-scheduling strategy for cytotoxic drug administration in cancer. It refers to frequent, regular administration of conventional chemotherapy at relatively subtoxic doses, with no prolonged break periods 1,3,4. In contrast to conventional chemotherapy, the primary target of metronomic chemotherapy is cancer vasculature, which plays a critical role in malignant tumour development and progression 1,5–7. In this context, it was initially considered and eventually shown that optimal antiangiogenic cancer therapy may steadily inhibit the proliferation and function of activated endothelial cells and therefore tackle cancer progression on a chronic basis 8.

Among several classes of cytotoxic substances, microtubule-targeted drugs are considered a reasonable choice for use in the setting of metronomic low-dose chemotherapy. They are known to act by inhibiting microtubule formation and function during mitosis, but they also have a potency to inhibit the function of endothelial cells at very low concentrations, a characteristic that is of major importance in metronomic chemotherapy 8,9. Vinorelbine (VRL) is a semisynthetic vinca alkaloid metabolized in the liver, the main metabolite of which, that is, 4-*O*-deacetylvinorelbine (DVRL), has been identified in the plasma of patients 10,11. The availability of an oral formulation of VRL (Navelbine Soft Caps) led to the clinical investigation of this drug as monotherapy at a metronomic dosing schedule, with promising results 10,12. In this setting, we investigated the functional and molecular effects of VRL and its metabolite DVRL against proliferating endothelial cells [human umbilical vein endothelial cell (HUVEC)] in an in-vitro simulation study of conventional and metronomic chemotherapy. Under the influence of protracted and short exposure to VRL, we studied cell growth and apoptosis and the effects on angiogenesis signalling mediators.

The aim of the current study was to assess whether protracted exposure of endothelial cells to VRL at low nanomolar and subnanomolar concentrations outperforms short exposure at high nanomolar to micromolar concentrations in inducing antiangiogenic effects at cellular and molecular levels. We also sought to investigate whether and to what extent experimental evidence matches the clinical experience with metronomic oral VRL, which yielded sustainable antitumor activity without overt toxicity 10,12.

## Materials and Methods

### Test compounds

Purified tartrate VRL and its active metabolite DVRL were kindly provided by the Institute de Recherché Pierre Fabre (Castres, France). Stock solutions were prepared (1 mmol/l, in conditioned medium), followed by filtration (0.2 μm, Whatman; Sigma Aldrich, Taufkirchen, Germany). Shortly before adding the research compounds to the cultured cells, each chemical was freshly diluted with conditioned medium to the desired concentration.

### Cell culture and treatment

HUVECs isolated from human umbilical cord veins, obtained from the Department of Gynaecology of the University Hospital of Ioannina, were digested with 0.1% type-I collagenase at 37°C for 12 min and cultured in M199 (Gibco, Gaithersburg, Maryland, USA) medium supplemented with 20% FBS, 30 mg/ml endothelial cell growth supplement (BD Biosciences, San Jose, California, USA), heparin (10 kU/ml; Sigma), l-glutamine and streptomycin/penicillin. Cells were used between the third and sixth passages. HUVECs were plated to subconfluence in plates coated with collagen (BD Biosciences) and treated with VRL for 4, 24 and 96 h. In the 96-h experiment, to simulate the metronomic dosing schedule, we replaced the drug-enriched medium every 24 h. The concentrations used in the metronomic model (0.001–100 nmol/l) were based on the results of pharmacokinetic analysis of two clinical studies, in conjunction with the half-maximal inhibitory concentrations (IC_50_) value of VRL in HUVECs. In particular, pharmacokinetic analysis of clinical trials on metronomic oral VRL showed that the steady-state trough levels of VRL averaged at 2–3 nmol/l 12. Therefore, up to these levels, the concentrations were considered metronomic. For this reason, we selected 10 nmol/l for nuclear and cytosolic protein extraction as the estimated *C*_max_ value of metronomic drug administration. In addition, concentrations between 100 nmol and 1 μmol/l simulate peak plasma levels of the conventional chemotherapy protocol, as specified by Marty *et al*. 13. Accordingly, we used a range of concentration between 0.1 nmol/l and 10 μmol/l to simulate the conventional bolus administration protocol, which included exposure of HUVECs to VRL/DVRL for 4 h, with a washout period with drug-free medium for the consecutive 92 h. At the end of exposure, both control and drug-treated cells were collected. Total RNA and protein fractions were extracted using the Nucleospin kit (Macherey-Nagel, Duren, Germany) according to the manufacture’s protocol and stored at −80°C. Medium supernatants were also collected, centrifuged at 1500 rpm/min and frozen at −80°C for enzyme-linked immunosorbent assay (ELISA) and pharmacokinetic analysis.

### Pharmacokinetic analysis

Conditioned medium obtained from control and drug-treated cells from the 24-h incubation with VRL/DVRL and the 96-h metronomic experiment (daily sampling, for 4 consecutive days) was shipped on dry ice to the Institute de Recherché Pierre Fabre (Castres, France), where it was analysed. Concentrations of VRL and DVRL were quantified using a sensitive liquid chromatography tandem mass spectrometry method that has been previously reported 14,15. The method has been used and validated for assessment of drug levels in human biological samples. For in-vitro systems a partial in-house validation has been performed at Centre de Recherche et Développement Pierre Fabre (personal communication, data not published).

### Proliferation assay

Proliferation inhibition testing was performed on HUVECs seeded in collagen-coated 96-well plates. Cells were harvested from cultures (at 80% confluence) by trypsinization, counted and plated at an optimal seeding density of 1000 cells/well. Cells were attached 24 h later; thereafter, the medium was replaced with growth medium containing the material to be tested and the cells were incubated at 37°C for 24 and 96 h in a 5% CO_2_ atmosphere. To maintain a constant concentration of the drugs during the protracted experimental period of 96 h, the medium was carefully removed every 24 h and fresh solution was added. Cell numbers were determined using a Cell Titter 96 AQueous One Solution Cell Proliferation (MTS) assay (Promega, Madison, Wisconsin, USA). Briefly, 20 μl of MTS solution was added to each well and the absorbance was recorded 2 h later. The results are expressed as a percentage of control cell proliferation, and IC_50_ values were assessed using Prism4 software (GraphPad Software Inc., La Jolla, California, USA).

### Apoptosis/cell-cycle analysis

To determine the proportion of apoptotic cells, the cells were treated with appropriate concentrations of the experimental drugs under the conditions described above. Thereafter, 5×10^5^ cells were diluted in 400 μl of the binding buffer, Annexin (included in the Annexin V-FITC kit, DK-700; Assay Designs, Ann Arbor, Michigan, USA). Part of the suspension (96 μl) was incubated with 1 μl Annexin V-FITC solution and 2.5 μl propidium iodide (250 μg/ml) on ice for 15 min in the dark, followed by dilution to 250 μl with the binding buffer; thereafter, apoptotic cells were counted by flow cytometry (Partec CyFlow ML, Germany).

To analyse cell cycle, both control and drug-treated cells were trypsinized, collected and fixed with cold 70% ethanol for 12 h at 4°C. After fixation, the cells were centrifuged at 1500 rpm/min, treated with 2 ml Cystain DNA 1 step/DAPI (PARTEC, Munster, Germany) at room temperature for 5 min and analysed using ultraviolet excitation (Partec CyFlow ML, Munster, Germany).

### qRT-PCR

To determine the mRNA expression of the angiogenesis-modulating genes CD36, cyclooxygenase-2 (COX-2), interleukin-8 (IL-8), peroxisome proliferator-activated receptor γ and thrombospondin-1 (TSP-1) we performed real-time quantitative reverse transcriptase-PCR (qRT-PCR) on a CFX96 Real-Time System (C1000 Thermal Cycler; Bio-Rad Laboratories, Hercules, California, USA), with IQ Supermix (Bio-Rad Laboratories), using specific primers/probe sets (Hs00354519_m1, Hs01573471_m1, Hs00174103_m1, Hs01115513_m1 and Hs00170236_m1, respectively; TaqMan Gene Expression Assays; Applied Biosystems, Foster City, California, USA).

For cDNA synthesis, 9 μl of total RNA (1 μg RNA) was reverse transcribed in 20 μl of reaction mixture containing 10 mmol/l dNTPs, 5× reaction buffer, a random hexamer primer [pd(N)_6_], 40 U/μl ribonuclease inhibitor and 20 U/μl reverse transcriptase (Fermentas, Sunderland, UK). The reaction mixture was incubated at 70°C for 3 min, chilled on ice for 5 min, and incubated at 37°C for 1 h; the reaction stopped with incubation at 70°C for 10 min. qRT-PCR was performed with the following amplification settings: 50°C for 2 min and 95°C for 10 min for the AmpliTaq activation; thereafter, 40 cycles of 15 s denaturation at 95°C, 15 s annealing at 60°C, 1 min extension at 60°C and melting.

### ELISA

A quantitative sandwich enzyme immunoassay technique was used to quantitate secreted IL-8 and TSP-1 (R&D Systems, Minneapolis, Minnesota, USA) in the conditioned medium obtained from control and drug-treated cells from the 96-h metronomic experiment. The concentration of proteins in unknown samples was determined by comparing the absorbance of the samples with the standard curve, according to the manufacturer’s protocol.

### Western blotting

Total cellular proteins were extracted and protein concentrations were determined using the suggested method for protein quantification in protein loading buffer PLB-TCEP using the NucleoSpin RNA/Protein isolation kit as described. Samples containing 10 μg of protein were analysed by 10% SDS-polyacrylamide gel electrophoresis, transferred onto nitrocellulose transfer membranes (Protran; Schleicher & Schuell, Dassel, Germany) and subjected to immunoblot analysis. Antibodies used in this study were as follows: PPARγ (sc-7273) and CD36 (sc-70642; Santa Cruz Biotechnology, Heidelberg, Germany), COX-2 (07-693; Upstate, New York, New York, USA) and a mouse monoclonal antibody to β-actin (CloneAC15, A5441; Sigma). Signals were revealed using horseradish peroxidase-conjugated secondary antibodies (Santa Cruz Biotechnology) and developed with an ECL detection kit (GE HealthCare, Little Chalfont, UK). Protein signals were normalized to the corresponding β-actin stain signal.

### Nuclear and cytosolic protein extraction

Nuclear and cytoplasmic extracts from HUVECs were prepared as previously described 16. Briefly, HUVECs plated in 10 cm dishes were grown to 80% confluence and treated with 10 nmol/l VRL for different time periods (0.5, 1, 3 and 16 h). Cells were collected and suspended in cold hypotonic buffer. After centrifugation, the cells were resuspended and lysed in hypotonic buffer containing NP-40 (0.1%). The cells were then centrifuged, and the cytoplasmic proteins were collected and stored (−80°C). The pellets were suspended in 50 μl ice-cold high-salt extraction buffer and incubated on ice for 30 min. The nuclear proteins were collected after centrifugation at 13000 rpm for 30 min at 4°C and stored at −80°C for further analysis.

Protein concentration was determined using the Bio-Rad Assay (Bio-Rad Laboratories). Cytoplasmic or nuclear extracts (10 μg) were resolved by 10% SDS-polyacrylamide gel electrophoresis, transferred onto nitrocellulose membranes and probed with rabbit polyclonal anti-nuclear factor (NF)-κB RelA/p65 (sc-372) and goat polyclonal anti-NF-κB p50 (sc-1190) as primary antibodies and lamin-B (sc-6216) or β-actin as secondary antibodies.

### Statistics

For statistical analysis, we used the GraphPad Prism4 software (GraphPad Software Inc.). Comparisons between groups were analysed using Student’s *t*-test. A probability value of less than 0.05 was considered statistically significant.

## Results

### Stability of VRL concentrations in tissue culture

**Fig. 1:**
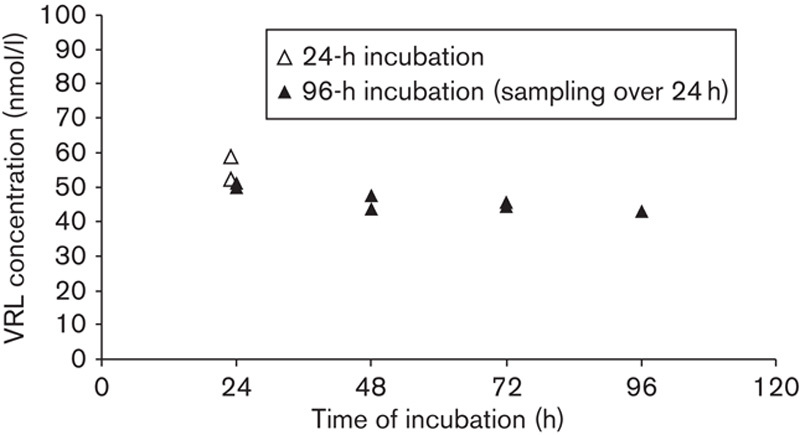
VRL concentrations after incubation of 100 nmol/l of substrate in HUVECs. Cells were exposed to VRL (100 nmol/l) for 24 h, as well as for the metronomic protocol of 96 h with daily medium change. Samples were collected at the end of the 24-h period. HUVEC, human umbilical vein endothelial cell; VRL, vinorelbine.

Drug analysis showed dose-related linearity for VRL levels in our in-vitro system (*R*^2^=0.98; Fig. S1, Supplemental digital content 1, *http://links.lww.com/ACD/A135*). In addition, in our metronomic model, the levels of VRL and DVRL in culture medium remained constant over the 96-h exposure period (Fig. 1 and Fig. S2, Supplemental digital content 2, *http://links.lww.com/ACD/A136*). For the active metabolite DVRL, mass spectrometry analysis revealed similar dose-related linearity to the parent compound (*R*^2^=0.9025; Fig. S1, Supplemental digital content 1, *http://links.lww.com/ACD/A135*), providing evidence that the active metabolite DVRL can be synthesized through VRL in HUVECs over a period of 24 h without accumulation. Overall, over the 96-h period, the conditioned medium had a constant concentration of both drugs, which meant in-vitro simulation of metronomic exposure.

### Effect of VRL on endothelial cell proliferation

**Fig. 2:**
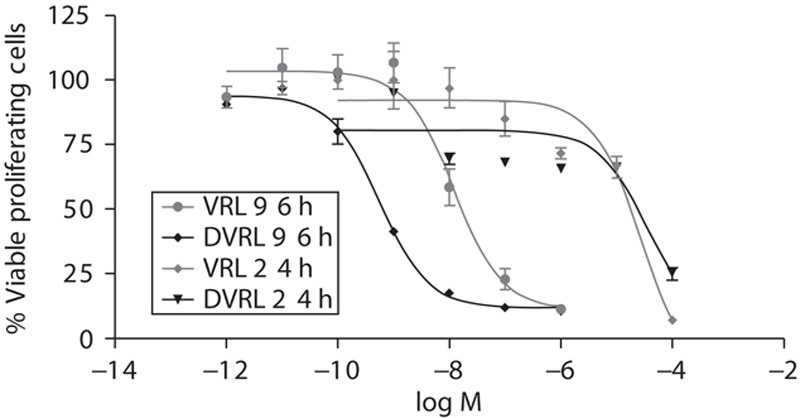
Inhibition of endothelial cell proliferation by VRL and DVRL. HUVECs were exposed to the indicated concentrations of VRL/DVRL for either 24 or 96 h. Viable cells were determined using an MTS assay. Results are expressed as a percentage of viable control cells and half-maximal inhibitory concentrations (IC_50_). DVRL, 4-*O*-deacetylvinorelbine; HUVEC, human umbilical vein endothelial cell; VRL, vinorelbine.

The IC_50_ at 96 h was four orders of magnitude lower compared with that for the 24-h exposure (1.23 nmol/l vs. 32 μmol/l for VRL and 0.55 nmol/l vs. 78 μmol/l for DVRL). Moreover, we found DVRL to be more active than VRL when considering endothelial cell growth (Fig. 2).

### VRL effects on cell-cycle distribution and apoptosis of HUVECs

**Fig. 3:**
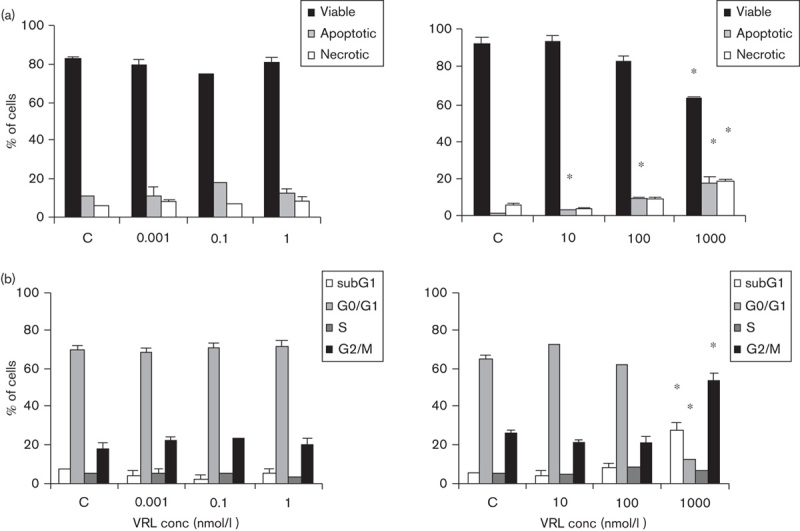
VRL on apoptosis and cell cycle. HUVECs were treated with VRL for 96 h (0.001, 0.1 and 1 nmol/l; metronomic model) or for 4 h, with a washout period of 92 h (10–1000 nmol/l; conventional chemotherapy). Percentages of (a) viable, total apoptotic and necrotic cells or (b) cells in cell-cycle phases in metronomic (left) versus conventional (right) chemotherapy were recorded. *P<0.05 compared with the control group. HUVEC, human umbilical vein endothelial cell; VRL, vinorelbine.

HUVECs were treated with VRL under the metronomic protocol over a concentration range from 0.001 nmol/l to 1 nmol/l for 96 h and under the conventional protocol at concentrations ranging from 10 nmol/l to 1 μmol/l for 4 h, followed by a washout period of 92 h. VRL treatment in the metronomic model (0.001–1 nmol/l) had no significant effect on cell-cycle progression and apoptosis, but short-term exposure at conventional concentrations induced both early and late apoptosis and necrosis; the dose of 1 μmol/l significantly increased the percentage of cells in the sub-G1 phase, decreased the number of viable cells and induced their accumulation in the G2-M cell-cycle phase (*P*=0.001; Fig. 3).

### Low-dose continuous exposure to VRL influences the expression of angiogenesis modulators at the mRNA level

**Fig. 4:**
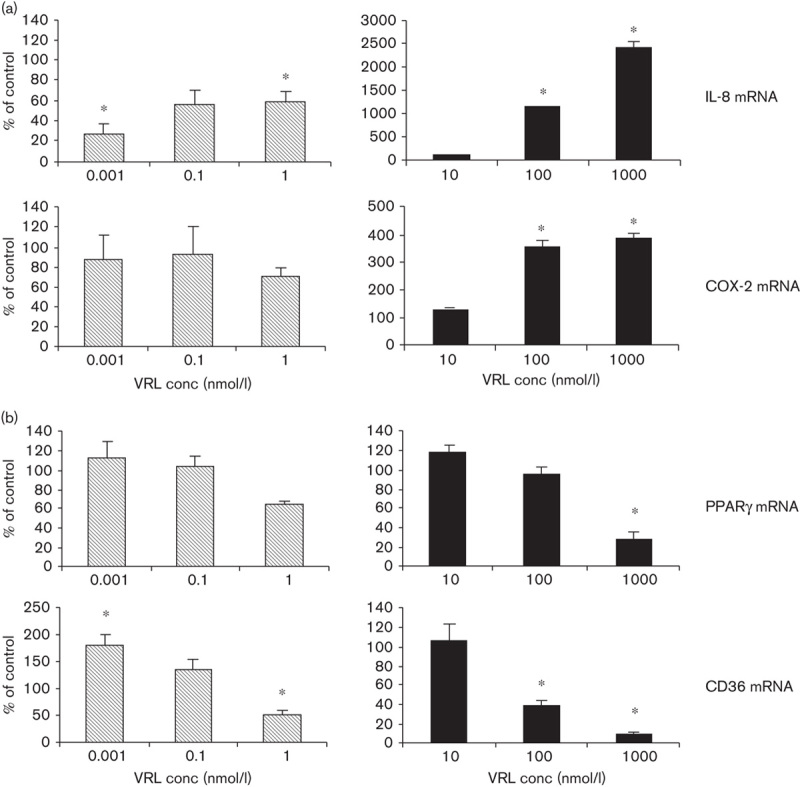
VRL on angiogenic markers. (a) Angiogenic and (b) antiangiogenic markers after applying the metronomic (left) or the conventional (right) chemotherapy *in vitro* model.*P<0.05 compared with the control group. VRL, vinorelbine.

qRT-PCR was carried out to investigate whether VRL could influence the synthesis of transcripts of angiogenesis modulators. Metronomic concentrations of VRL led to dose-dependent decreases in proangiogenic (IL-8) and increases in antiangiogenic genes (PPARγ and CD36; Fig. 4). Protracted exposure to a low concentrations of VRL (0.001, 0.1 or 1 nmol/l) suppressed mRNA levels of IL-8 (*P*<0.05) but did not affect COX-2 mRNA levels (Fig. 4a). However, expression of both genes was induced when the cells were exposed to VRL concentrations above 10 nmol/l on using the conventional chemotherapy protocol (Fig. 4a).

For PPARγ, low concentrations of VRL (0.001 and 0.1 nmol/l) had no effect on basal levels; however, the lowest tested dose of 0.001 nmol/l induced the expression of CD36 (*P*<0.05) during the metronomic schedule of 96 h of exposure (Fig. 4b). In contrast, concentrations higher than 10 nmol/l caused a significant reduction in the mRNA of antiangiogenic molecules such as PPARγ (*P*<0.05 at 1 μmol/l) and CD36 (*P*<0.05 at 100 nmol/l; Fig. 4b).

### Decrease in IL-8 and increase in PPARγ secreted proteins on metronomic exposure of HUVECs to VRL

**Fig. 5:**
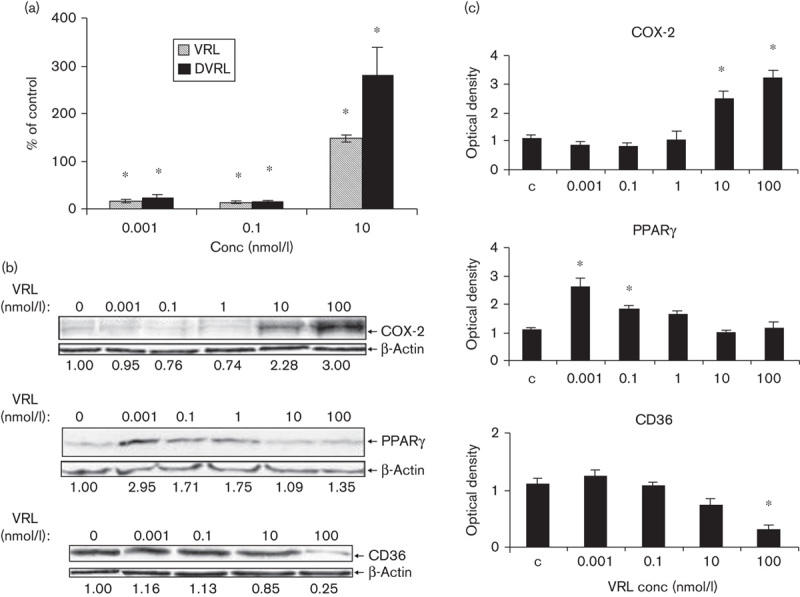
VRL/DVRL on angiogenic and antiangiogenic proteins in the protracted administration model. (a) IL-8 protein levels were determined by ELISA. (b) Protein levels of COX-2, PPARγ and CD36 were determined by western blotting using respective antibodies; the last blot shows only the nonglycosylated but full-length CD36 protein (55 kDa). The numbers under the blots indicate values normalized to β-actin values of the respective proteins. Representative figures of two independent experiments are shown. *P<0.05 compared with control group. COX, cyclooxygenase; DVRL, 4-*O*-deacetylvinorelbine; HUVEC, human umbilical vein endothelial cell; IL, interleukin; PPARγ, peroxisome proliferator-activated receptor γ; VRL, vinorelbine.

Similar effects were seen on the protein level following exposure of HUVECs to metronomic concentrations of VRL. Under the metronomic schedule, 0.001 and 0.1 nmol/l VRL diminished secreted IL-8 protein levels (*P*<0.05; Fig. 5a) and increased PPARγ protein levels (three times the control levels; Fig. 5b). Protein expression of COX-2, and CD36, was similar to that of control. However, higher concentrations of VRL (10 and 100 nmol/l) either had no effect on antiangiogenic proteins, for example, PPARγ, or lowered CD36 levels (*P*<0.05; Fig. 5b). This range of concentrations also increased angiogenic COX-2 protein expression (Fig. 5b) and secreted IL-8 protein concentration (Fig. 5a). As far as DVRL is concerned, we observed that DVRL had the same activity profile as VRL, for IL-8 (Fig. 5a).

### Involvement of NF-κB in VRL-induced IL-8 and COX-2 transcription

**Fig. 6:**
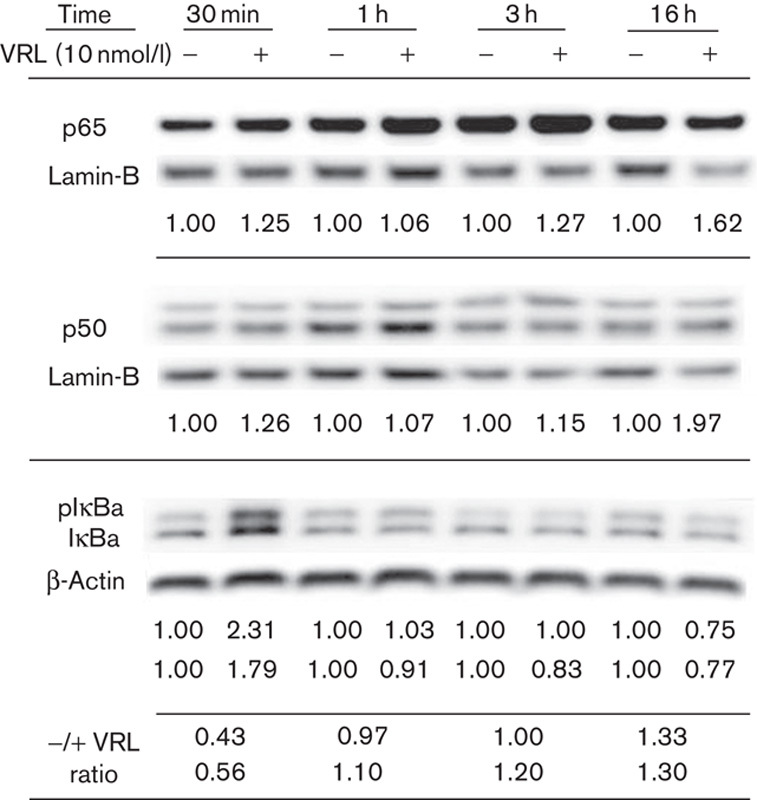
Activation of the NF-κB pathway by VRL. Both the p65 and the p50 subunits were determined in the nuclear extracts of HUVECs (normalized to lamin-B). IκBa proteins were determined in the cytosol and normalized to β-actin. The presented values express the protein levels of NF-κB units normalized to the respective housekeeping protein before and after VRL treatment; for IκBa, values are for pIκBa and IκBa, respectively. The last two lines correspond to pIκΒa/ IκΒa ratio before and after VRL treatment at each time point. The upper line corresponds to pIκBa and the lower line to IκBa. Representative figures of two independent experiments are shown. HUVEC, human umbilical vein endothelial cell; NF-κB, nuclear factor-κB; VRL, vinorelbine.

To determine the potential involvement of transcriptional factor NF-κB in VRL-induced IL-8 and COX-2 signalling pathways, cytosolic and nuclear extracts were isolated from HUVECs after a time-course of 0.5 to 16 h of exposure to 10 nmol/l VRL, which was the estimated *C*_max_ value of metronomic VRL in the clinical setting. (Fig. 6). Western blot analysis was carried out for NF-κB subunits of p65 and p50 and for IκBa; expression was calculated after normalization to lamin-B or β-actin. Protein expression of both NF-κB subunits was increased in the nucleus even at 30 min, and it remained elevated until 16 h after VRL addition. A translocation of p65 and p50 to the nucleus, accompanied by elevated levels of phosphorylated and nonphosphorylated IκBa 17, was observed during the first 30 min after VRL treatment. This effect was minimized at 1 h, followed by degradation of pIκBa and IκBa. The ratio values for both forms (before and after VRL) became greater than 1.0 over time (Fig. 6).

## Discussion

Metronomic oral VRL was been shown in clinical studies to yield sustainable antitumor activity in a number of metastatic cancers, possibly through an antiangiogenic mechanism 10,12,18. We sought to investigate the cellular and molecular effects of metronomic VRL and DVRL on HUVECs, in an attempt to elucidate the underlying mechanisms of their antiangiogenic activity. We found that metronomic exposure of HUVECs to VRL/DVRL halts the growth of endothelial cells at low nanomolar concentrations and induces molecular effects that favour the expression of antiangiogenic modulators and suppresses the expression of proangiogenic modulators.

Metronomic chemotherapy is, by concept, an endothelial cell-targeted antiangiogenic therapy that exploits the high turnover rate and remarkable sensitivity of endothelial cells to cytotoxic agents 7. For this reason, we selected HUVEC cultures, which are considered an acceptable model of proliferating endothelial cells. With regard to the selection of the most appropriate drug concentrations, we took into consideration the pharmacokinetic analysis data of clinical studies 10,12. In this context, we considered determination of drug levels in our in-vitro study to be important to ensure that conclusions could be relevant to the clinic. We found that the levels of VRL in the culture medium remained constant over the 96-h exposure period in our model, which is in line with clinical data that showed no evidence of accumulation over time. We understand, as limitations of this work, the fact that we opted not to include tumour cells in this study to focus on the effects of metronomic VRL on endothelial cells, and the relatively limited number of angiogenesis modulators selected for investigation, which, however, are among the most common, with some being therapeutic targets on their own 17,19,20.

One of the important steps in antiangiogenesis is the inhibition of endothelial cell proliferation. In our in-vitro metronomic model, the IC_50_ of VRL was 1.23 nmol/l. This concentration is lower and possibly not representative of clinically observed metronomic trough drug levels in the clinical studies 10,12. Interestingly, these drug concentrations did not have an obvious effect on apoptosis or the cell cycle. In contrast, in the study arm that was compatible with conventional chemotherapy, the IC_50_ was much higher (∼30 μmol/l) and these concentrations produced G2-M cell-cycle arrest and induced apoptosis.

With regard to angiogenesis modulators, we found that metronomic and conventional chemotherapy models influenced the basal levels of IL-8 and PPARγ in a dose-dependent manner, but in the opposite manner. Characteristically, metronomic VRL was found to decrease both mRNA and secreted protein levels of IL-8, whereas conventional treatment produced a marked increase in IL-8 mRNA. Keeping IL-8 at low levels is potentially important in cancer therapy. Characteristically, overexpression of IL-8 has been associated with tumour progression and metastasis 21, and in a clinical trial of metronomic oral VRL, patients who benefited from this therapy tended to have low levels of IL-8 10. A pathway closely related to IL-8 is that of COX-2, an enzyme that stimulates angiogenesis. Low metronomic concentrations of VRL did not affect the expression of COX-2, but high concentrations over 96 h of treatment activated COX-2 protein production. In contrast, metronomic concentrations of VRL that reduced IL-8 levels caused induction of protein expression of the antiangiogenic PPARγ. Concentrations of VRL above 10 nmol/l, which increased angiogenic molecules (IL-8 and COX-2), minimized the production of the PPARγ protein. The antiangiogenic TSP-1 receptor CD36 was not affected by metronomic treatment of HUVECs, whereas conventional treatment decreased both mRNA and protein levels of CD36.

Previous studies have indicated that PPARγ, COX-2 and IL-8 signalling pathways are regulated by NF-κB 19,22. A recent study showed the involvement of NF-κB in VRL-induced oxidative injury, but with doses higher than those used in typical chemotherapy 23. In our study, VRL at a concentration of 10 nmol/l caused instant rapid activation of the NF-κB pathway, as shown by p65/p50 nuclear translocation and IκBa release, which indicates potential involvement of NF-κB in affecting IL-8 and COX-2 upregulation and PPARγ deactivation by VRL concentrations just above the steady-state trough levels of metronomic dosing.

### Conclusion

Protracted exposure of endothelial cells to VRL at very low concentrations that simulate steady-state serum levels achieved with chronic metronomic administration of oral VRL has shown superiority compared with short high-dose exposure that simulates conventional pulse dosing of VRL in inducing antiangiogenic effects at cellular and molecular levels. This study provides experimental evidence that supports recent favourable clinical findings of metronomic oral VRL and suggests potential therapeutic combinations that could be of clinical interest.

## Supplementary Material

SUPPLEMENTARY MATERIAL
