# Supplementary figures and images for: Cellular and molecular effects of metronomic vinorelbine and 4-O-deacetylvinorelbine on human umbilical vein endothelial cells
Source: Anticancer Drugs. 2016 Feb 1;27(3):216–24. doi: 10.1097/CAD.0000000000000319 (PMC4733659; doi:10.1097/CAD.0000000000000319)

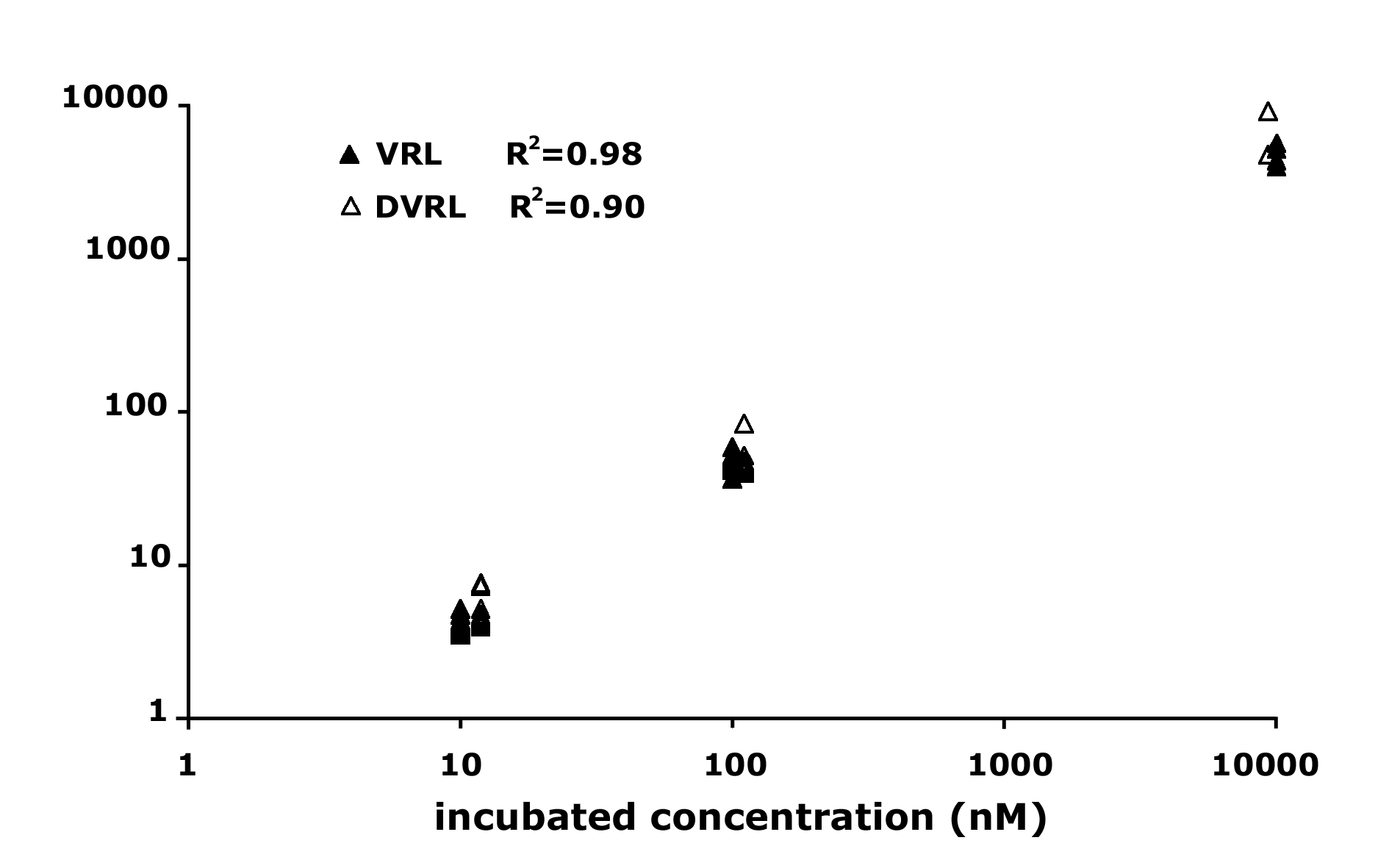

Supplement: SUPPLEMENTARY MATERIAL [file cad-27-216-s001.tif]

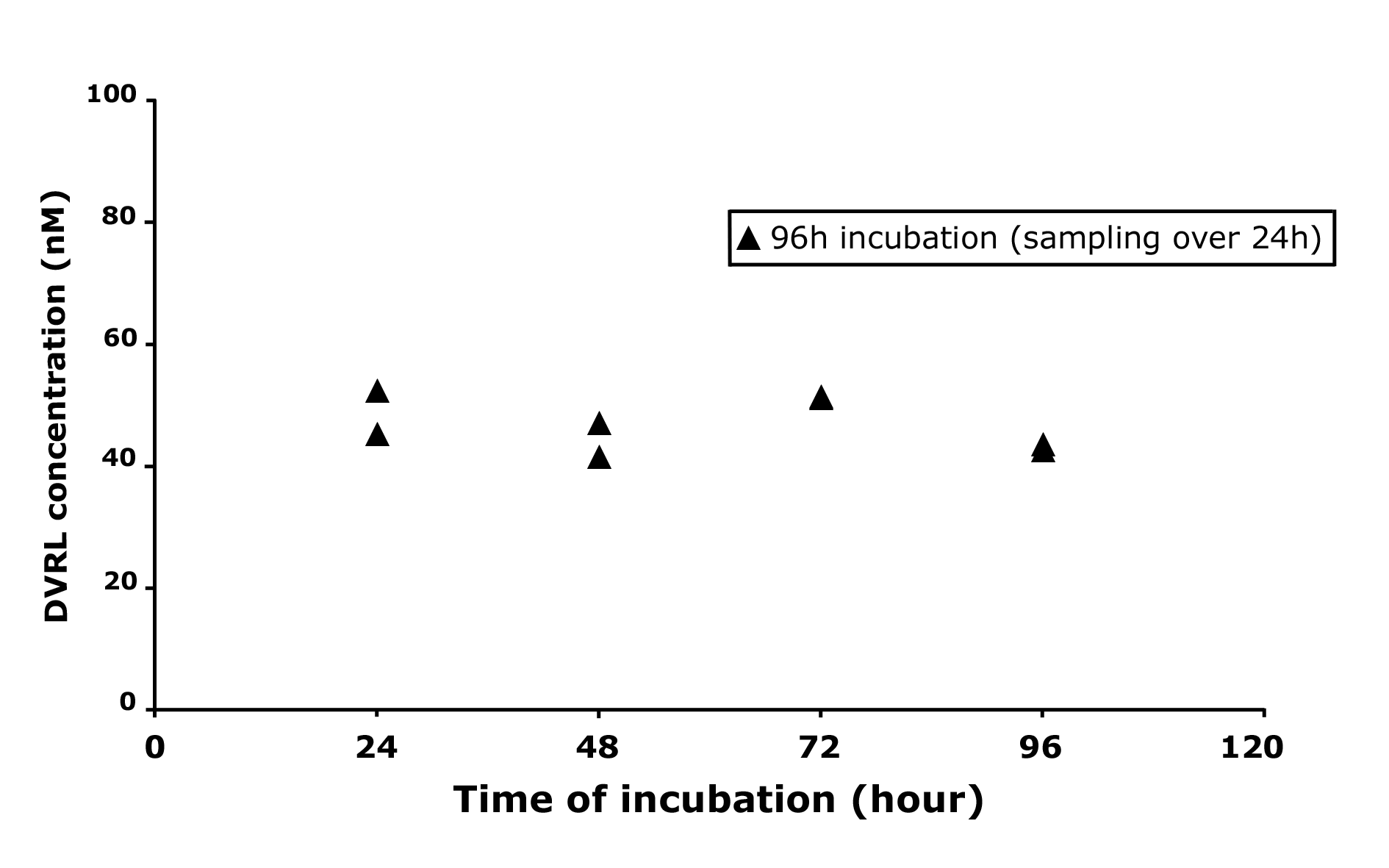

Supplement: SUPPLEMENTARY MATERIAL [file cad-27-216-s002.tif]
